# Intersections of ageism and gender stigma: Exploring long-term care employees' attitudes towards aging

**DOI:** 10.1093/geroni/igab046.3408

**Published:** 2021-12-17

**Authors:** Tarah Loy-Ashe, Brent Hawkins

**Affiliations:** 1 Southern CT State University, New Haven, Connecticut, United States; 2 University of North Carolina Wilmington, Wilmington, North Carolina, United States

## Abstract

The purpose of this mixed methods, single case study was to explore long-term care (LTC) employees’ attitudes towards age and gender. The intersection of Rosemary Garland-Thomson’s theory of feminist disability (2001) and Hailee Gibbons’ compulsory youthfulness theory (2016) provided the conceptual framework for this project. The sample consisted of 60 LTC direct care employees, all employed at the same organization, who completed an on-line survey during the COVID-19 pandemic. The survey consisted of demographic questions and the Fraboni Scale of Ageism. Twenty-one of these employees participated in a 30-45 minute phone interview. Cultural artifacts were also collected. All data were collected during the COVID-19 pandemic. Although quantitative results showed no statistical significance, qualitative results suggest that employees do exhibit some ageist attitudes towards the residents for whom they care. Despite evidence that most employees felt a deep connection with residents, they detached themselves from the aging process. The theoretical framework lends hand in explaining how both ableism and ageism appeared to contribute to this detachment. Findings indicated employees’ interactions and attitudes towards residents were influenced by themes compassionate ageism, ableism, and identity, which resulted in meta theme caregiver validation and reward. Employees received validation and altruistic reward from positive interactions with what they perceived to be “ideal” residents; those who fit the stereotype of a nursing home resident, such as older, pleasant, and dependent. The perceived “ideal” residents varied by gender. Generally, female residents were expected to be more independent and at times viewed negatively when requesting assistance.

